# The Political Legitimacy of the Healthcare System in Portugal: Insights from the European Social Survey

**DOI:** 10.3390/healthcare9020202

**Published:** 2021-02-13

**Authors:** Maria Asensio

**Affiliations:** CIES-IUL, ISCTE—University Institute of Lisbon, 1649-026 Lisboa, Portugal; Maria.asensio@iscte-iul.pt

**Keywords:** political legitimacy, healthcare, austerity measures, Portugal

## Abstract

This article investigates the political legitimacy of the health care system and the effects of austerity on the population’s welfare, paying particular attention to Portugal, a country severely harmed by the economic crisis. Based on analysis of data collected from the European Social Survey on 14,988 individuals living in private households during the years between 2002 and 2018, the findings of this study aim to analyze the social and political perception of citizens on the state of health services in two distinctive periods—before and after the economic crisis, according to self-interest, ideological preferences, and institutional setup as predictors of the satisfaction with the health system. The results demonstrate a negative attitude towards the health system over the years, a consistent drop during the financial crisis period, and a rapid recovery afterward. The research also shows that healthcare evaluations depend on the perceived institutional effectiveness in the citizenry’s eyes. The more the citizens perceive the government as effective and trust-worthy, the more they are satisfied with the health system. Also, differences in healthcare evaluations among social groups were felt unequally: while vulnerable citizens were more affected by the Government’s plan of austerity measures for health reform, healthcare evaluations of better-off social groups—younger individuals, those with higher incomes, higher education, and better health status—did not decline. This study contributes to the academic debate on the effects of austerity on the population’s welfare attitudes and highlights the need to examine the different impacts of reforms introduced by the crisis on social groups.

## 1. Introduction

The study of popular support to healthcare has long been a critical feature of the welfare states and has increasingly become the subject matter of empirical research on opinions, values, and attitudes [1,2,3,4]. The empirical literature provides ample evidence that public health has always been welcomed and supported, showing a recurrent pattern of popularity across nations, even in periods of welfare state retrenchment [5,6,7,8,9]. These differences raise the question of what makes individuals be satisfied with the health care system over time? How can we explain the influence of financial and economic crises on public opinion towards healthcare? One of the significant findings is that individuals’ perceptions are not driven by self-interest motives and ideology alone. According to this approach, a lack of satisfaction with the healthcare system leads to increased distrust of government institutions and a loss of legitimacy in citizens’ eyes, creating a general feeling that governments are not responding to citizens’ needs and expectations. Thus, politicians’ perceived responsiveness can be a crucial determinant of citizens’ political trust, especially their trust in the health care system.

Three general explanations have been proposed in the literature. First, citizens may be moved by self-interest. The self-interest approach rests on the assumption that citizens support healthcare because they benefit from access to public health and have health services provided by the State. Second, support for the healthcare system may result from a broad set of ideological beliefs influencing their attitudes on social, political, and economic matters [10]. Here, it is theorized that individuals proclaim support for healthcare because they support the political values and principles as the basis of the welfare state institution [11,12]. Third, citizens’ perceptions are influenced by the institutional characteristics of the health system. It is argued that different types of healthcare systems generate different attitudinal patterns. As explained by Rothstein and Steinmo [13], citizens pay attention to the procedures they experience when interacting with the National Health System institutions.

This article presents research paths that expand the current academic debate on a broad set of institutional, ideological, and personal factors that may influence public attitudes towards the Portuguese health system across time. Based on data from the European Social Survey (ESS) from 2002 to 2018, the findings of this study aim to analyze the social and political perception of citizens on the state of health services in two distinctive periods—before and after the economic crisis, according to self-interest, ideological preferences, and institutional setup as predictors of the satisfaction with the health system. This research strategy allows us to contribute to the ongoing discussion on the effects of the crisis on public opinion towards Portugal’s healthcare system before and after the crisis. This study helps to understand better the evolution overtime of satisfaction with the healthcare system. Therefore, by an empirical study on how efficiently the State manages public resources and how responsive the political system is to citizens’ preferences, this study aims to fill this gap. These results are explained in the context of Portugal’s national health system. Concerning broad welfare policies, there is a rich body of literature analyzing Western welfare states’ legitimacy. Many previous works have found that self-interest, ideological orientation, and institutional characteristics of the healthcare system are vital determinants of citizens’ attitudes towards the healthcare system [14,15,16,17,18]. While these analyses have illuminated many sources of modern welfare states’ legitimacy, there are two significant shortcomings. First, with a few exceptions [2,19], most previous studies focused on the entire social policy regime. Simultaneously, little understanding has been gained on the factors that explain attitudes towards the healthcare system, a vital component of the welfare regime. Second, most previous studies that focus on citizens’ perceptions tend to neglect the variable of perceived economic conditions [20]. Hence, this article analyzes individuals’ attitudes towards the healthcare system before and after the economic crisis by focusing on their perceptions about economic conditions and government effectiveness rather than objective assessment related to attitudes towards specific healthcare system aspects. This study makes three contributions. First, it extends to Portugal the discussion on the popular legitimacy of the welfare state in Western Europe. Second, an understanding of health policy legitimacy is adopted, and the study reveals the factors associated with such legitimacy. Third, this study is intended to provide health policymakers with evidence-based recommendations that consider the citizens’ views.

## 2. Theoretical Background: Citizens’ Support of the Health Care System and Predictors

The study of public opinion, values, and attitudes on healthcare has increasingly become the subject matter of empirical research [3,21]. The first studies emerged in the context of the legitimacy crisis during the mid-1970s. Coughlin’s research showed that even though healthcare services vary among countries, public attitudes were invariably popular, and individual citizens supported health care principles and accepted its requirements and outcomes. Rose and Peters [22] worried that support would decline due to economic recession and rising demand for health care schemes. Wilensky [23] and Galbraith [24] proved the detrimental effects of the spending pressure that arises from the expansion of the welfare state and the conflict created between those dependent on the welfare state and those who perceive themselves as taxpayers and assuming the burden of the welfare state that would eventually lead to the withdrawal of popular support in Western welfare states [25].

In the 1990s, new theories refuted this line of argument with a new approach based on normative beliefs and social values as the basis of a particular ideal of social justice represented in welfare policies. This legitimacy defined by norms that are valued and accepted by citizens provided a factual motivational foundation for supporting health care provision [26,27]. Additionally, empirical analyses found strong and constant popular support for the health care systems in most Western countries [12,14,28]. Building on earlier work [21,29] the current literature has reconsidered the importance of why citizens show different levels of commitment to the welfare state but also why these attitudes differ between other social groups [9,30]. In particular, the relevance of five broad sets of predicting attitudes in healthcare evaluation have been explained according to (i) self-interest; (ii) current economic situation; (iii) ideological preferences; (iv) institutional performance; (iv) social trust.

### 2.1. The Self-Interest Hypothesis

According to this hypothesis, those citizens who benefit from the welfare state tend to demonstrate more significant support and have a positive assessment of public health care arrangements. Age, income, education, sex, and health status are often used to test the self-interest thesis. This thesis maintains that older, sick, low-income, low-educated, unemployed, and pensioned individuals are typically associated with higher health risks and fewer financial resources, and they are therefore to be more supportive of public health care arrangements [16,21,29]. However, there is the counter-argument according to which the better-off individuals—those with higher economic status and better health—might also support the healthcare system because of normative attitudes, such as the expectations towards significant support to the state’s role in welfare provision [31]. However, Gevers et al. [32], for example, argued that a preference for state provision of health care in Europe was stronger among those in poor health, but no effects were found from age or education. Missinne et al. [19] found that being lower-income and female tended to demonstrate more significant support. Insights concerning satisfaction were also mixed. Some authors [33,34] claim that people with lower socioeconomic status tend to receive less medical care, resulting in less satisfaction, while others maintain that as this group of people have lower expectations on the health system, they are not certainly dissatisfied [2,19,35]. A reasonably consistent example is the association between poor health and low satisfaction, implying that frequent visits to healthcare services tend to reduce the level of satisfaction [2,35,36].

### 2.2. Economic Performance

The challenges that national health systems face today are mostly performance-related. While support for the public health system remains universally high and stable in Portugal, political disaffection is increasing, especially since the onset of the financial crisis in 2008 [7]. So, how can we account for the Portuguese trend in satisfaction with the health system? As Portugal was under the International Monetary Fund (IMF) conditionality, people suffered considerably from consequences of the great recession; the literature mainly attributes the decreasing levels of satisfaction to the persistent economic crisis [10,37]. That economic conditions impact the health system is a common argument in comparative politics, and many scholars see economic performance as crucial for satisfaction with the health system. From this perspective, an economic crisis is likely to undermine citizens’ satisfaction with health care provision. When the economy is going badly, citizens are far more critical about the health system’s performance. However, is there an effect of the economy on public evaluations of the health care system, or is this issue more salient during crisis times? Can we observe similar relationships at the individual level? Several studies have shown that people’s perceptions of the past, present and current state of the economy shape their evaluations of their public health system’s functioning. Similarly, the economic well-being of citizens appears to be a good predictor of satisfaction: more prosperous, working individuals who evaluate their financial situation favorably tend to be more satisfied with the health care system than more impoverished, unemployed respondents [38,39,40].

### 2.3. The Ideological Thesis

Ideology emerged as the second set of predictors from cross-national comparative studies. This theoretical argument sustains no association between citizens’ socioeconomic status and attitudes towards social welfare because their ideological orientation mediates this effect. The ideology of individuals determines their opinions regarding the degree of support to the health system [12,33,41]. Several studies have shown how political values and ideologies are connected to healthcare systems’ opinions, how such values affect attitudes and perceptions toward specific healthcare policies, and how voters’ opinion formation can be affected by elite-driven partisan polarization and framing appeal specific political values. According to Gevers et al. [32] left-oriented European citizens are more likely to support the role of the State in healthcare issues and recognize a strong association of individuals’ political attitudes with healthcare satisfaction [5]. The authors argue that healthcare satisfaction is associated with higher levels of political trust [34]. On the other hand, socio-political values are an essential predictor of citizens’ support of health care systems: principles of equality or solidarity are defended by those who support public healthcare organizations. Some studies have revealed that individuals in favor of the egalitarian tenets are the most generous supporters of public intervention in healthcare systems but, on the other hand, egalitarians are not the more satisfied with the system [19].

### 2.4. Political Trust Thesis

Theories of legitimacy point to another explanation for varying levels of support in the health care system: political trust in democratic institutions [12,42,43]. Political trust is included to explore the extent to which the health system satisfaction may be associated with trust in political institutions more broadly, with respondents asked to indicate the extent to which most people personally can be trusted, people try to be helpful. They trust in politicians and the legal system. These theories assume that trust in institutions is based on legitimacy and that political trust is associated with political participation and consent. People who trust that political power is appropriately exercised will give more support to policy efforts. Citizens who do not believe that they can trust their government are less likely to express support for the health care system. Trust, however, must not be seen as identical to legitimacy, but rather “as one of the dynamics of public opinion, helping to explain citizens’ attitudes and actions vis-a-vis the regime” [42].

## 3. The Portuguese National Health System

### 3.1. A Dual Health Care System

The Portuguese health system is characterized by a National Health System created in 1979 to promote universal coverage for all health needs, and the state budget finances it. Under a complementary regime, the health system is provided by social and private insurance schemes with which the state contracts a set of services to improve the citizens’ access to health care [44]. Taxes finance the health system through the state budget. During this period, the Portuguese healthcare system also witnessed a series of changes to improve the system’s performance [45]. These changes involved the introduction of public–private partnerships in the hospital sector, implementation of a more effective purchaser-provider split, promotion of generic medicines, reduction of medicine prices, reorganization of the public network of healthcare services and reform of primary care that involved the creation of Family Health Units and the creation of a network for long-term care [46]. Until 2013, specific social groups could have access and receive health care benefiting from additional public funding through either social insurance schemes based on occupancy or voluntary private health insurance schemes, which generated inequality with other citizens. This benefit no longer happens today, and the public subsystem is now fully financed by its beneficiaries [47]. From a macro perspective, the health system is delivered by a mix of public and private health care providers generating inequality, in which specific better-off social groups enjoy better access and conditions of care, while low-income families such as the unemployed, the old, the poor and the disabled do not have equal access to health care [7].

### 3.2. The Impact of Austerity Measures on the Portuguese National Health System

The economic and financial crisis in Portugal led to external intervention by the International Monetary Fund, the European Commission, and the European Central Bank through the signing of a memorandum of understanding (MoU) [48]. The austerity policies imposed by the financial assistance program of the Troika aimed to halt or reduce public expenditure and generated a series of cuts in public spending and effects justified by the risk of a failure to meet the goals defined in MoU that had a significant impact on the lives of the citizens. The economic crisis had a direct consequence on health expenditure. According to the *Instituto Nacional de Estadística* (INE) (2014), current expenditure on health in 2013 continued to decline (−2.1%), but less intensely than in 2011 (−5.2%). Between 2010 and 2012, the public expenditure reduced 27% in staff expenses and reduced 81% of capital expenses between 2010 and 2014 [49].

The MoU consisted of a large set of measures directed explicitly to the health sector with a negative impact on social equality. In the specific area of health, the Memorandum includes the following measures: (i) strict control of costs in the health sector with a significant reduction in operating costs, expenses with overtime compensation and transport costs for patients; (ii) the increase in user charges for National Health System (NHS) users or moderating fees (user fees) in parallel with a stricter design of tax exemption means-testing criteria; (iii) the substantial cut in tax deductions for health, including private insurance (by two-thirds in total); (iv) the reduction in the budgetary cost of health benefit schemes for civil servants; (v), reducing drug reimbursement to patients [50].

The drastic application of the Memorandum measures generated a series of effects that significantly impacted Portuguese citizens’ lives. The effect of the crisis and the damages caused by austerity to the Portuguese economy and society manifested in many ways: employment destruction; precariousness of the younger segments of the population; large emigration flow of qualified workers; worsening of poverty, social exclusion, and income inequalities. The social impact of austerity felt unequally on families and individuals [51].

### 3.3. Consequences of Crisis and Austerity on Inequalities

According to a report of the European Commission (2013), one of the consequences of the austerity programs based on regressive taxes and spending cuts threatened to dismantle the mechanisms that reduce inequality and enable equity growth. Therefore, evidence shows that the prominent supporters of austerity, such as the International Monetary Fund, recognize that harsh austerity measures have not brought the expected results and harmed growth and equality [52].

Even more, studies show that with the implementation of the Troika’s austerity measures, the Portuguese population had a reduction in the economic power of citizens, the NHS lost responsiveness and increased barriers to access, expanded families’ out-of-pocket health spending, reduced efficiency and quality of service provision and minimized public healthcare investment [53].

Despite the measures intended to protect the most vulnerable, such as user fees exemptions, several studies have shown that the duplication of the amount of moderating fees and the extension of the moderating fees to other services, along with the increase of the delay to access healthcare due to the scarcity of professionals, have aggravated the situation namely for those patients who cannot afford to use the private sector. The crisis has interfered with access to healthcare, whether the dimensions considered are those associated with supply or those associated with demand [54].

### 3.4. Predictors of Health Policy Legitimacy

These findings will confirm that the austerity measures introduced into the Portuguese healthcare system during the crisis affected the healthcare evaluation of the general evaluation of particular social groups. The scale of austerity measures implemented after 2011 (MoU) [50] were much higher than the scale of the initial crisis measures (until 2011) and more strongly affected public healthcare services users. Unable to sustain its public finances, Portugal’s government requested the International Monetary Fund and the European Union a full bailout package in 2011. Therefore, the government was impelled to adopt severe austerity measures during those years, producing a decline in many citizens’ standard of living. With the introduction of the Memorandum measures, it was expected that healthcare evaluations of individuals would have dropped more strongly than before the economic crisis.

## 4. Data, Variables and Methods

### 4.1. Data

**Figure 1:**
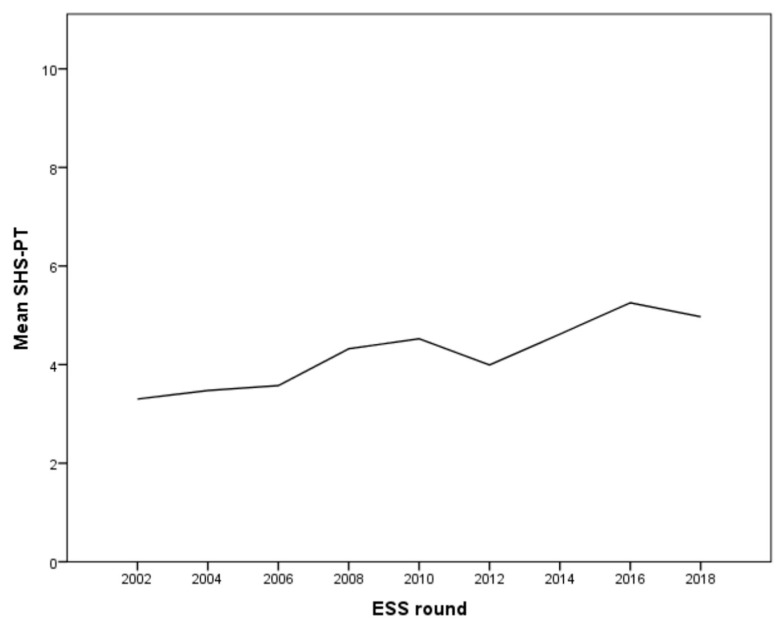
Mean satisfaction level of healthcare services in Portugal, ESS, 2002–2018.

This article explored empirical data from the European Social Survey (ESS) to ascertain what shifts, if any, may be evident in people’s attitudes to political legitimacy in Portugal since the impact of the 2011 economic crisis and subsequent political changes. The ESS is a multi-national survey that began in 2002 and has been repeated every two years since that date. Portugal has participated in all ESS rounds and covers 16 years (2002–2018). While response rates vary from a low of 6.6% in Round 9 (2017–2018) to14.8% in Round 4 (2007–2008), the ESS aims to interview a nationally representative sample on questions regarding media usage, social exclusion, socio-political orientations, and attitudes, values, religiosity, identity, attitudes towards immigrants and socio-demographics for every round of the survey. The purpose of this study was to assess the evaluation of the state of health services in Portugal from 2002 to 2018, considering a set of independent variables aggregated according to five different models. All models were set to be adjusted by sex and age. Because the ESS is a cross-sectional survey, a new probability sample is drawn in each ESS round. After checking the trend of evaluating the healthcare system and the rounds, it was observed a non-linear trend, most likely due to the financial crisis from 2010 to 2012 (Figure 1). Hence, all analysis was stratified due to the advantage that exists in adjusting models with different behavior between different temporal strata, considering the separation 2002–2010 versus 2012–2018.

### 4.2. Variables

The dependent variable is the evaluation of the state of health services in Portugal. Respondents were asked what they “think overall about the state of health services in their country nowadays” on an 11-point state, ranging from 0, extremely bad, to 10, extremely good. Time dummy variables for each year measure changes in the evaluation of healthcare services across time. To explore subgroup differences in healthcare evaluations, we include demographic and socio-economic characteristics of the respondent, such as the respondent’s sex (1 = female, 0 = male) and age (<21, 21–35, 36–49, 50–64, 65+ years). To test for health needs, we included two health variables: the respondent’s self-reported health status measured on a 5-point scale, ranging from very good to good, fair, bad, and very bad health; and health-related limitations in daily lives and routines re-coded into a dummy variable (1 = a lot/to some extent; 0 = no limitations). Since information on household income is missing for wave 5 in the Portuguese dataset, we use a subjective income variable as a proxy for the financial resources available to the household. Respondents were asked how they feel about their household income nowadays and whether they live comfortably on the current income, cope on present income, or find it very difficult to live on the current income.

### 4.3. Method

Statistical analysis was performed under SPSS, version 24. Descriptive statistics were presented as means (M) and standard deviations (SD) for continuous variables with symmetric distributions and medians (P25–P75) otherwise. Categorical variables were presented as frequencies (n) and percentages (%).

Simple linear regressions were implemented to screen predictors for the state of health services in Portugal. Significant variables were included in multiple linear regression. Estimates were calculated according to the minimum ordinal square’s method. The model adjustment was assessed with F test to assess the predictors’ contribution to the outcome. Residuals normality was checked with the Shapiro-Wilk test, confirming this assumption (*p* > 0.05). Standardized residuals were calculated to observe outliers’ inexistence (ri < |3|), and no outliers were found. Homoscedasticity was checked following the standardized residuals versus standardized predicted values plot, confirming that no significant results were found. Finally, multicollinearity was assessed with VIF (<4); VIF results showed no multicollinearity evidence.

## 5. Results

### 5.1. Sample

Our sample included 14,988 respondents who were enquired on nine rounds, from 2002 to 2018 in Portugal. This study measured changes in the evaluation of healthcare services using time dummy variables from 2002 to 2018. In order to explore subgroup differences in health care evaluations, there were included demographic and socio-economic characteristics such as sex (0 = male, 1 = female) and age (<21, 21–35, 36–49, 5–64, 65+ years).

### 5.2. Evaluations of Healthcare Services in Portugal

In line with previous research, Portuguese citizens are very critical of their health services. Figure 1 provides an overview of healthcare services’ mean satisfaction level in Portugal between 2002 and 2018, a country that belongs to European countries with the most negative health services evaluations. With a mean of 3.30 (SD = 2.13), Portuguese residents rated their healthcare services very negatively in 2002. Since then, evaluations had increased steadily until 2010, when evaluations reached a mean of 4.52 (SD = 2.17). With the signing of the Memorandum and critical measures’ performance, evaluations dropped to 3.99 (SD = 2.28) in 2012. Despite the ongoing reform processes and the implementation of remaining Memorandum measures until 2015, healthcare evaluations increased again in 2015 to 4.62 (SD = 2.56), slightly above the value reached in 2011/12. Still, in 2018, healthcare evaluations dropped to a mean of 4.08 (SD = 2.31).

### 5.3. Evaluation of Healthcare Services in Portugal across Time

**Figure 2:**
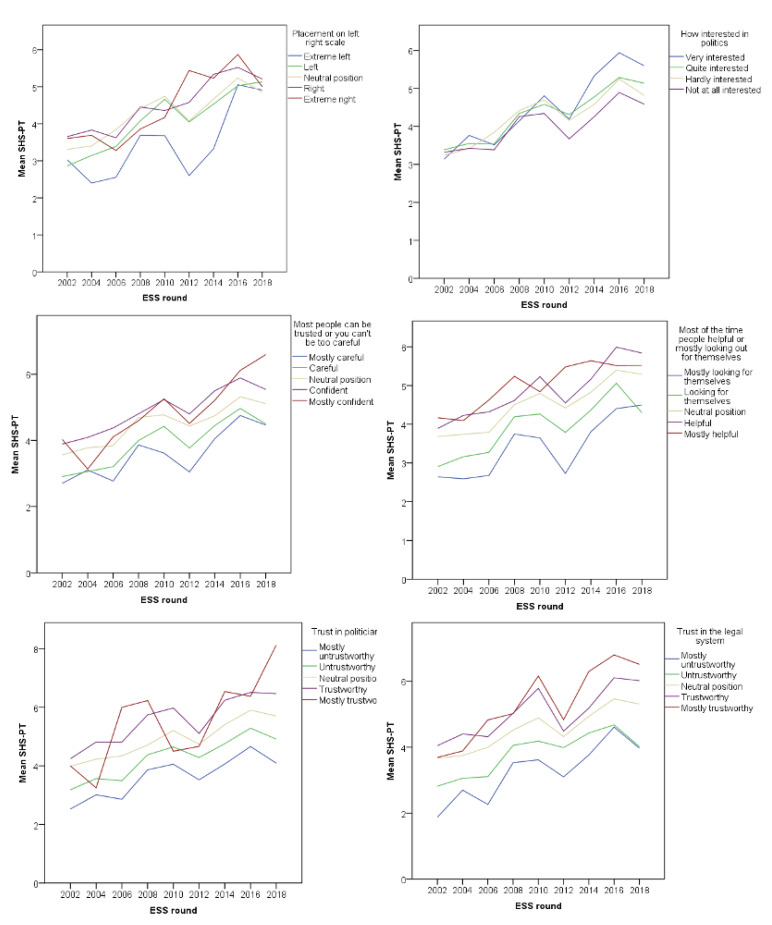
Relationship of Healthcare mean satisfaction levels and support for the role of government.

**Figure 3:**
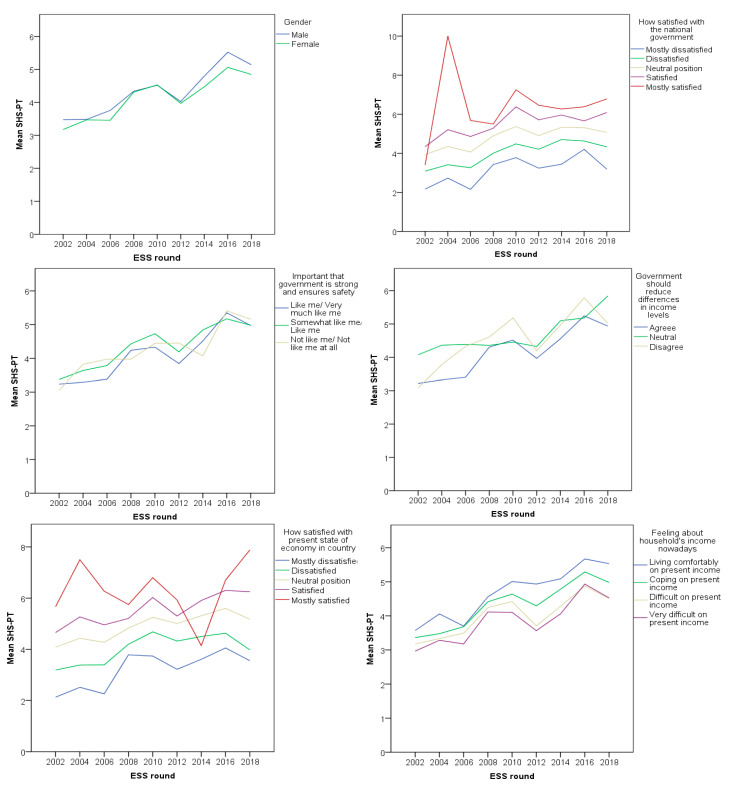
Relationship of ideological disposition, interest in politics, social trust, and satisfaction with the healthcare system in Portugal.

**Figure 4:**
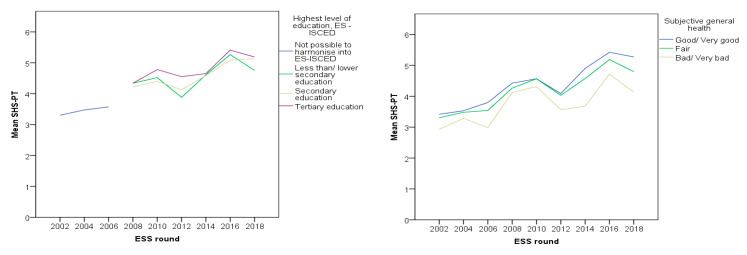
Perception of the healthcare considering education and subjective general health.

Figure 2, Figure 3 and Figure 4 show the trend of healthcare evaluation in association with all proposed models, in all nine rounds of the ESS. In a general manner, there is a constant dissatisfaction over the years, with a consistent drop around 2010–2012, during the financial crisis period. In the Portuguese case, the mean values of satisfaction across all subjective income groups seem to attenuate the healthcare perception drop during the financial crisis period and a rapid recovery afterward. Individuals who place themselves on the right are more satisfied with the healthcare system and are more likely to be protected from the crisis perception. On the contrary, those individuals who place themselves on the left side are less satisfied with the healthcare system and tend to exacerbate their perception of the health system. According to the newest scale, the research did not measure education level from 2002 to 2006; nevertheless, higher education seems to attenuate crisis perception regarding healthcare evaluation. 

### 5.4. Healthcare Evaluations Associations

Table 1 (model 1) shows that the level of satisfaction with the health system depends on the perceived institutional effectiveness in the eyes of citizenry. The more the citizens perceive government as effective and trustworthy, the more they are satisfied with the health system. On the contrary, left wing citizens have more negative attitudes toward the health system because they vary with incumbent support. Left-wing citizens were dissatisfied with the Passos Coelho government and, therefore, also with the health system. Stratified results show a positive linear trend for β coefficients on satisfaction with government association with healthcare evaluations. Results for 2012–2018 strata showed higher coefficients for all categories, namely satisfied (β = 2.43; *p* < 0.001) and mostly satisfied (β = 3.06; *p* < 0.001), suggesting a recovery of healthcare evaluation after 2012. Government strength and safety (somewhat like me/ like me) revealed a positive association with the perception of the healthcare evaluation for 2002–2010 strata (β = 0.28; *p* < 0.001). The same happened for the 2012–2018 strata with the positive association for responses “somewhat like me/like me” (β = 0.16; *p* = 0.013). Neutral responses regarding government policy for income equity were more positively associated (β = 0.33; *p* < 0.001) than disagreement responses (β = 0.25; *p* = 0.036) for strata 2002–2010; on the contrary, for the 2012–2018 strata, no significant results were found for neutral responses and disagreement were positively associated (β = 0.36; *p* = 0.029) with healthcare evaluation. Compared with males, females were negatively associated with the perception of healthcare system on the 2012–2018 strata (β = −0,27; *p* < 0.001); this result was consistent with all models.

When adjusting for the stratification variable (Table 2), the study found a positive effect for the perception of the healthcare system in 2012–2018 (β = 0.61; *p* < 0.001), suggesting better results from 2012–2018. Females continued to be negatively associated with the evaluation of the healthcare system (β = −0.14; *p* < 0.001). Satisfaction with government presented a linear trend of its coefficients towards the upper end of the scale, namely “satisfied” (β = 2.18; *p* < 0.001) and “mostly satisfied” (β = 2.65; *p* < 0.001). A significant association was also found for “somewhat like me/ like me” responses (β = 0.24; *p* < 0.001), concerning government strength and safety. Government policy for income equity was associated with increased perception of the healthcare system for responses towards neutral responses (β = 0.29; *p* < 0.001) and disagreement of equity policies (β = 0.29; *p* = 0.003).

Table 3 (model 2) shows the association of the current economic situation with the state of health services in Portugal. Survey respondents were asked how they “feel about their household income nowadays” and whether they live comfortably on present income, cope on present income, find it difficult to live on present income, or find it very difficult to live on present income. Age had a positive impact on the perception of evaluation of the healthcare system for the strata 2002–2010. This result was consistent with all models and strata; nevertheless, the effect size is quite negligible, near 0 for all models. People that are more satisfied with the economy tend to have a better perception of the healthcare system, especially after 2012. However, an impressive result was observed for those who felt most satisfied with the economy between 2002–2010, with a very high effect size (β = 3.21; *p* < 0.001), compared to the respondents with the same opinion in 2012–2018 (β = 2.80; *p* < 0.001). Negative opinions about the household income had a higher impact on the perception of evaluation of the healthcare system on the strata 2012–2018, especially for those who felt difficulties on the current income (β = −0.76; *p* < 0.001) and felt very difficulties on the current income (β = −0.70; *p* < 0.001). The same effect was felt in 2002–2010, but lower on the effect size.

When adjusting for the stratification variable (Table 4) in the model measuring the impact of the current economic situation, the study found a positive effect for the perception of healthcare evaluation on 2012–2018 (β = 0.56; *p* < 0.001), suggesting better results for these strata. Satisfaction with economy showed a linear trend associated with the perception of healthcare evaluation, namely regarding normal positions (β = 1.60; *p* < 0.001) and especially those who felt satisfied (β = 2.32; *p* < 0.001) and mostly satisfied with the economy (β = 2.88; *p* < 0.001). Negative feelings about household income maintained their linear trend of growing negative perception on the evaluation of the healthcare system for those who felt more difficulties but reached a plateau at the last levels: coping on present income (β = −0.32; *p* < 0.001), difficult on present income (β = −0.50; *p* < 0.001) and very difficult on present income (β = −0.46; *p* < 0.001).

Table 5 (model 3) shows that citizens’ ideological preferences (left-oriented or right-oriented) may offer some clues regarding their attitudes toward the healthcare system. The coefficients show that right-wing citizens tend to show higher levels of satisfaction with the healthcare system. In fact, political mindsets tending to right were increasingly associated with a better perception of the healthcare system, particularly for 2012–2018, namely right (β = 1.15; *p* < 0.001) and extreme right (β = 1.46; *p* < 0.001). Before the economic crisis (2002–2010), left-oriented citizens were less satisfied with health services (β = 0.59; *p* < 0.001) than those citizens positioned in a centrist position (β = 0.92; *p* < 0.001) or those right-oriented citizens: β = 0.91; *p* < 0.001). After the economic crisis, ratings increased particularly for right-oriented citizens (β = 1.15; *p* < 0.001) and extreme-right oriented citizens (β = 1.46; *p* < 0.001). Interestingly, interest in politics was not significant for the perception of the healthcare system from 2002 to 2010, but this changed in 2012–2018: decreasing interest in politics was associated with a more critical perception of the healthcare system, with ratings for those quite interested (β = −0.47; *p* < 0.001), hardly interested (β = −0.70; *p* < 0.001) and not at all interested (β = −1.15; *p* < 0.001).

After adjusting for the stratification variable (Table 6) in the model measuring the impact of ideological preferences, the study found a positive effect regarding their attitudes toward the evaluation of the healthcare system on 2012–2018 (β = 0.83; *p* < 0.001), suggesting better results from 2012–2018; this was the highest impact of all five models. Political mindsets towards right were associated with positive perceptions of the healthcare system, namely right (β = 1.01; *p* < 0.001) and extreme right (β = 1.04; *p* < 0.001). Decreasing interest in politics was linearly associated with a lower perception of the healthcare system, quite interested (β = −0.23; *p* <0.001), hardly interested (β = −0.28; *p* < 0.001) and not at all interested (β = −0.55; *p* < 0.001).

Table 7 (model 4) shows the results for the impact of social trust in the perception of healthcare evaluation. Respondent’s confidence about people trustworthiness was found to be positively associated with the perception of the healthcare system, especially for the strata 2012–2018, confident (β = 0.71; *p* < 0.001) and mostly confident (β = 0.75; *p* = 0.017), but also for the strata 2002–2010, neutral position (β = 0.35; *p* < 0.001) and confident (β = 0.52; *p* < 0.001). Respondent’s opinion about people helpfulness was positively associated with the healthcare system’s perception, exhibiting a linear trend for both strata. For 2002–2010, respondents that consider politicians to be helpful scored, on average, more than 0.76 (*p* < 0.001) on the healthcare evaluation scale, and more 1.13 (*p* = 0.002) for those who consider them to be most trustworthy. For strata 2012–2018 results were 0.97 (*p* < 0.001) and 1.33 (*p* < 0.001), respectively. Trust in politicians followed the same trend, with increased results of perception of the healthcare system for those that considered politicians to be more trustworthy, on both strata. Finally, regarding trust in the legal system, results of the healthcare system perception were higher for those who trust more, but this time in the 2002–2010 strata, except for those who consider a politician is to be most trustworthy; in this case, the highest result was for the 2012–2018 strata (β = 1.71; *p* < 0.001).

When adjusting for the stratification variable (Table 8) in the model measuring the impact of social trust, the study found a positive effect for the perception of the healthcare system in 2012–2018 (β = 0.72; *p* < 0.001), suggesting better results from 2012–2018. People’s trustworthiness was associated with a higher perception of the healthcare system, for responses classified as neutral position (β = 0.38; *p* < 0.001), confident (β = 0.60; *p* < 0.001), and most confident (β = 0.35; *p* < 0.001). People’s helpfulness, trust in politicians, and trust in the legal system showed a linear trend for the association with the health system’s perception, with significant results for all categories (*p* < 0.001).

Finally, Table 9 (model 5) shows results for the association of self-interest evaluating the healthcare system. This model was particularly challenging because of the available data for the highest level of education. In the first three ESS rounds (2002, 2004, and 2006), data was classified as “not possible to harmonize.” So, for the strata 2002–2010, the study obtained other results from 2008–2010. It resulted in different reference categories for the variable: “not possible to harmonize” for 2002–2010, and “less than secondary education” for 2012–2018. Despite these issues, conclusions stand for a higher perception of the health system considering increasing education degrees, especially for the 2002–2010 strata, maybe because of the referred data bias. On the other hand, a wrong or very bad perception of one’s health tends to have a negative impact on the perception of the healthcare system: β = −0.45 (*p* < 0.001) for 2002–2010 and β = −0.88 (*p* < 0.001) for 2012–2018.

In this model, the strata variable was significant (β = 0.13; *p* = 0.004), suggesting that the perception of the healthcare system was higher for 2012–2018 strata, considering education and perception of subjective general health. Positive and negative linear trends, respectively, were found for the coefficients of the highest level of education and subjective general health regarding the association with the healthcare system’s perception, suggesting a higher perception of the healthcare system among the more educated and healthier individuals (Table 10).

## 6. Discussion

Since the economic crisis of 2011, there has been an overall decrease in satisfaction with the healthcare system, influenced by the austerity measures such as the reduction of public spending and the increase of tax revenues to reduce the budget deficit. This result is in line with previous findings of Missinne [19] and Kohl and Wendt [55] who concentrated on the EU countries to explain that the legitimacy of government institutions is a prerequisite for citizens to be satisfied with these welfare institutions. While there is still scarce evidence on how the health sector cuts affected the Portuguese population’s experiences with the healthcare system, this study suggests that the crisis effect was particularly strong for the vulnerable groups. Based on the analysis of the data collected from the European Social Survey between 2002 and 2018, this study’s findings demonstrate a low level of satisfaction with the state of the health system.

The study focused the analysis on examining the predictors of healthcare satisfaction. The self-interest argument and the ideology argument were considered in the analysis; the government’s trust was also considered as a potential explanatory variable. Multivariate analysis suggested that the healthcare system’s perception was higher for 2012–2018 strata, considering education and perception of subjective general health. Age had a positive impact on the perception of evaluation of the healthcare system for the strata 2002–2010. However, poor health status and negative feelings about household income maintained their linear trend of growing negative perception on the healthcare system’s evaluation for those who felt more difficulties.

The ideology argument received more decisive empirical confirmation. In Portugal, healthcare satisfaction declined among the left-oriented between 2010 and 2012, and right-side political ideas tended to protect from the crisis perception. Our results provide support for the moderating influence of ideology on self-interest. In Portugal, in the most vulnerable group, between 2010 and 2012, satisfaction dropped for the left-oriented but increased for the right-oriented, and these changes were greater than the changes in the vulnerable groups. Our findings show that between 2010 and 2012, the satisfaction of left-oriented Portuguese citizens decreased more than those on the right. These findings suggest that the degree of satisfaction on left-oriented citizens was more affected by the Government’s plan of austerity measures for health reform that were introduced after the signing of the Memorandum. It is not guaranteed that the degree of criticism that is attributed to the health system was greater with the economic crisis than that encountered in the past. While the critical climate during the Troika agreement looks like a significant disjuncture with the past, there are also grounds to confirm the mainstream argument that ideology is at the core of health policy opinion formation [46]. Healthcare is a political issue and is different from other social policies because of its more universal implications. Nearly all citizens directly experience how it operates, what authority, why it operated, and whose interests it represents. Therefore, this is a sphere where strong solidarity is usually observed. Another significant finding is that trust of the political institutions seems to reinforce health policy legitimacy. Citizens’ trustworthiness was associated with a higher perception of the healthcare system [56]. People’s helpfulness, trust in politicians, and trust in the legal system showed a linear trend for the association with the health system’s perception, with significant results for all categories. This study provides new evidence that trust in the government is significantly associated with higher satisfaction and with more support for the governmental provision of healthcare. So, as a result, this study needed to assess both the direction and extent of change and identify whether the driving force behind the institutional and political levels or more in the social and cultural context against which health systems operate.

## 7. Robustness Checks and Limitations

In interpreting the results, it is significant that the results remain robust when we use coefficients that are statistically significant in a variety of models. The fact that these data replicate and strongly reflect the pattern found previously—using similar comparisons of health care evaluations of the general population in the same country—provides considerable confidence in the stability and consistency of attitudinal indicators that stand alongside existing behavioral and factual indicators of Portuguese well-being and the robustness of the measures employed. The application of quantitative methods to study complex social phenomena such as citizens’ attitudes requires some simplification and abstraction. However, what makes operationalization possible could place limitations on the interpretation of our findings: the most notable refers to the known weaknesses associated with the use of population satisfaction as a measure of health system performance. There are external factors to the health system that can impact satisfaction but have little value in determining performance levels, and population satisfaction produces levels of ambiguity that can make policy recommendations and changes more difficult. Another limitation is that, unlike longitudinal analyses with repeated observations of the same individual, where the estimates are averages of changes over time of each respondent, our estimates are calculated as differences over time in group averages. Finally, another limitation is that there were different measurement forms in the schooling variable along waves. The study did not measure the education level from 2002 to 2006 according to the newest scale; nevertheless, higher education seems to attenuate crisis perception regarding the state of health systems in Portugal.

## Figures and Tables

**Table 1:** Political responsiveness (Model 1).

	2002–2010 R^2^ = 0.304; F = 194.93 (*p* < 0.001)				2012–2018 R^2^ = 0.313; F = 169.55 (*p* < 0.001)			
	β	SE	*p*	95% CI	β	SE	*p*	95% CI
Socio-Demographic Characteristics Female (0 = male)	−0.07	0.04	*p* = 0.099	−0.16; 0.01	−0.27	0.06	*p* < 0.001 *	−0.40; −0.15
Age	≈0	≈0	*p* = 0.610	−0.01; 0.01	≈0	≈0	*p* = 0.827	−0.01; 0.01
Institutional Effectiveness Satisfaction with government								
Mostly dissatisfied	-	-	-	-	-	-	-	-
Dissatisfied	0.71	0.06	*p* < 0.001 *	0.60; 0.82	1.04	0.09	*p* < 0.001 *	0.87; 1.21
Neutral position	1.46	0.06	*p* < 0.001 *	1.35; 1.57	1.78	0.09	*p* < 0.001 *	1.63; 1.94
Satisfied	2.00	0.10	*p* < 0.001 *	1.79; 2.20	2.43	0.11	*p* < 0.001 *	2.21; 2.65
Mostly satisfied	2.09	0.25	*p* < 0.001 *	1.60; 2.57	3.06	0.20	*p* < 0.001 *	2.67; 3.45
Government strength and safety								
Like me/Very much like me	-	-	-	-	-	-	-	-
Somewhat like me/Like me	0.28	0.04	*p* < 0.001 *	0.19; 0.36	0.16	0.06	*p* = 0.013 *	0.03; 0.28
Not like me/Not like me at all	0.07	0.11	*p* = 0.498	−0.14; 0.28	0.30	0.16	*p* = 0.053 *	−0.04; 0.60
Government policy for income equity								
Agree	-	-	-	-	-	-	-	-
Normal	0.33	0.08	*p* < 0.001 *	0.17; 0.49	0.17	0.13	*p* = 0.209	−0.10; 0.43
Disagree	0.25	0.12	*p* = 0.036 *	0.02; 0.48	0.36	0.17	*p* = 0.029 *	0.04; 0.69

* statistically significant.

**Table 2:** Model 1—Political responsiveness adjusted for stratification variable.

Predictors	β	SE	*p*	95% CI
Socio-Demographic Characteristics Female (0 = male)	−0.14	0.04	*p* < 0.001 *	−0.21; −0.07
Age	≈0	≈0	*p* = 0.729	−0.01; 0.01
Institutional Effectiveness Satisfaction with government				
Mostly dissatisfied	-	-	-	-
Dissatisfied	0.82	0.05	*p* < 0.001 *	0.73; 0.92
Normal position	1.58	0.05	*p* < 0.001 *	1.49; 1.67
Satisfied	2.18	0.07	*p* < 0.001 *	2.03; 2.33
Mostly satisfied	2.65	0.15	*p* < 0.001 *	2.25; 2.94
Government strength and safety				
Like me/Very much like me	-	-	-	-
Somewhat like me/Like me	0.24	0.04	*p* < 0.001 *	0.17; 0.31
Not like me/Not like me at all	0.16	0.09	*p* = 0.071	−0.01; 0.33
Government policy for income equity				
Agree	-	-	-	-
Neutral	0.29	0.07	*p* < 0.001 *	0.15; 0.43
Disagree	0.29	0.10	*p* = 0.003 *	0.10; 0.48
Stratification				
2002–2010	-	-	-	-
2012–2018	0.61	0.04	*p* < 0.001 *	0.53; 0.68

* statistically significant; R^2^ = 0.129; F = 370.53 (*p* < 0.001).

**Table 3:** Model 2—current economic situation.

Predictors	2002–2010 R^2^ = 0.284; F = 214.28 (*p* < 0.001)				2012–2018 R^2^ = 0.130; F = 207.39 (*p* < 0.001)			
	β	SE	*p*	95% CI	β	SE	*p*	95% CI
Socio-Demographic Characteristics Female (0 = male)	−0.07	0.04	*p* = 0.115	−0.16; 0.02	−0.21	0.07	*p* < 0.001 *	−0.33; −0.08
Age	0.01	≈0	*p* = 0.001 *	0.01; 0.01	0.01	≈0	*p* = 0.096	−0.01; 0.01
Satisfaction with economy								
Mostly dissatisfied	-	-	-	-	-	-	-	-
Dissatisfied	0.78	0.05	*p* < 0.001 *	0.67; 0.88	0.88	0.09	*p* < 0.001 *	0.71; 1.04
Neutral position	1.50	0.06	*p* < 0.001 *	1.38; 1.61	1.75	0.08	*p* < 0.001 *	1.59; 1.91
Satisfied	2.10	0.14	*p* < 0.001 *	1.83; 2.38	2.49	0.14	*p* < 0.001 *	2.22; 2.76
Mostly satisfied	3.21	0.37	*p* < 0.001 *	2.50; 3.93	2.80	0.30	*p* < 0.001 *	2.22; 3.38
Subj. Household income (0 = living comfortably)								
Living comfortably on present income	-	-	-	-	-	-	-	-
Coping on present income	−0.19	0.08	*p* = 0.024 *	−0.36; −0.03	−0.41	0.10	*p* < 0.001 *	−0.61; −0.22
Difficult on present income	−0.26	0.09	*p* = 0.003 *	−0.44; −0.09	−0.76	0.11	*p* < 0.001 *	−0.97; −0.55
Very difficult on present income	−0.25	0.10	*p* = 0.015 *	−0.45; −0.05	−0.70	0.13	*p* < 0.001 *	−1.00; −0.44

* statistically significant.

**Table 4:** Model 2—current economic situation adjusted for stratification variable.

Predictors	β	SE	*p*	95% CI
Socio-Demographic Characteristics Female (0 = male)	−0.12	0.04	*p* = 0.001 *	−0.19; −0.05
Age	0.01	≈0	*p* < 0.001 *	0.01; 0.01
Satisfaction with economy				
Mostly dissatisfied	-	-	-	-
Dissatisfied	0.82	0.05	*p* < 0.001 *	0.74; 0.90
Normal position	1.60	0.05	*p* < 0.001 *	1.51; 1.69
Satisfied	2.32	0.10	*p* < 0.001 *	2.14; 2.51
Mostly satisfied	2.88	0.23	*p* < 0.001 *	2.44; 3.33
Subj. Household income (0 = living comfortably)				
Living comfortably on present income	-	-	-	-
Coping on present income	−0.32	0.06	*p* < 0.001 *	−0.45; −0.30
Difficult on present income	−0.50	0.07	*p* < 0.001 *	−0.63; −0.36
Very difficult on present income	−0.46	0.08	*p* < 0.001 *	−0.61; −0.20
Stratification				
2002–2010	-	-	-	-
2012–2018	0.56	0.04	*p* < 0.001 *	0.49; 0.64

* statistically significant; R^2^ = 0.118; F = 409.93 (*p* < 0.001).

**Table 5:** Model 3—ideological preference.

Predictors	2002–2010 R^2^ = 0.08; F = 10.81 (*p* < 0.001)				2012–2018 R^2^ = 0.038; F = 46.30 (*p* < 0.001)			
	β	SE	*p*	95% CI	β	SE	*p*	95% CI
Socio-Demographic Characteristics Female (0 = male)	−0.19	0.05	*p* = 0.001 *	−0.29; −0.08	−0.23	0.07	*p* = 0.001 *	−0.37; −0.09
Age	0.01	≈0	*p* = 0.043 *	0.01; 0.01	0.01	0.01	*p* = 0.372	−0.01; 0.01
Left-Right Schema								
Extreme left	-	-	-	-	-	-	-	-
Left	0.59	0.14	*p* < 0.001 *	0.32; 0.86	0.70	0.15	*p* < 0.001 *	0.41; 0.98
Centrist neutral position	0.92	0.13	*p* < 0.001 *	0.66; 1.17	0.77	0.13	*p* < 0.001 *	0.52; 1.03
Right	0.91	0.14	*p* < 0.001 *	0.64; 1.18	1.15	0.15	*p* < 0.001 *	0.85; 1.44
Extreme right	0.65	0.18	*p* < 0.001 *	0.31; 1.00	1.46	0.18	*p* < 0.001 *	1.10; 1.82
Interest in politics								
Very interested	-	-	-	-	-	-	-	-
Quite interested	≈0	0.11	*p* = 0.988	−0.21; 0.21	−0.47	0.13	*p* < 0.001 *	−0.72; −0.22
Hardly interested	0.07	0.11	*p* = 0.505	−0.14; 0.29	−0.70	0.13	*p* < 0.001 *	−0.95; −0.44
Not at all interested	−0.09	0.11	*p* = 0.385	−0.31; 0.12	−1.15	0.13	*p* < 0.001 *	−1.41; −0.89

* statistically significant.

**Table 6:** Model 3—ideological preferences adjusted for stratification variable.

Predictors	β	SE	*p*	95% CI
Socio-Demographic Characteristics Female (0 = male)	−0.20	0.04	*p* < 0.001 *	−0.28; −0.11
Age	0.01	≈0	*p* = 0.014 *	0.01; 0.01
Political mindset				
Extreme left	-	-	-	-
Left	0.64	0.10	*p* < 0.001 *	0.45; 0.84
Centrist position	0.88	0.09	*p* < 0.001 *	0.70; 1.10
Right	1.01	0.10	*p* < 0.001 *	0.82; 1.21
Extreme right	1.04	0.13	*p* < 0.001 *	0.80; 1.29
Interest in politics				
Very interested	-	-	-	-
Quite interested	−0.23	0.08	*p* = 0.005 *	−0.40; −0.07
Hardly interested	−0.28	0.08	*p* = 0.001 *	−0.44; −0.12
Not at all interested	−0.55	0.08	*p* < 0.001 *	−0.72; −0.39
Stratification				
2002–2010	-	-	-	-
2012–2018	0.83	0.04	*p* < 0.001 *	0.74; 0.91

* statistically significant; R^2^ = 0.044; F = 108.66 (*p* < 0.001).

**Table 7:** Model 4—social trust.

Predictors	2002–2010 R^2^ = 0.100; F = 174.52 (*p* < 0.001)				2012–2018 R^2^ = 0.127; F = 132.75 (*p* < 0.001)			
	β	SE	*p*	95% CI	β	SE	*p*	95% CI
Socio-Demographic Characteristics Female (0 = male)	−0.09	0.04	*p* = 0.047 *	−0.17; −0.01	−0.24	0.06	*p* < 0.001 *	−0.37; −0.12
Age	0.01	≈0	*p* = 0.037 *	0.01; 0.01	0.01	≈0	*p* = 0.005 *	0.01; 0.01
People trustworthiness								
Mostly careful	-	-	-	-	-	-	-	-
Careful	0.08	0.07	*p* = 0.232	−0.05; 0.22	0.09	0.10	*p* = 0.373	−0.11; 0.29
Neutral position	0.35	0.07	*p* < 0.001 *	0.21; 0.48	0.42	0.09	*p* < 0.001 *	0.24; 0.61
Confident	0.52	0.09	*p* < 0.001 *	0.34; 0.70	0.71	0.12	*p* < 0.001 *	0.47; 0.95
Mostly confident	0.06	0.18	*p* = 0.742	−0.29; 0.40	0.74	0.23	*p* < 0.001 *	0.29; 1.19
People helpfulness								
Mostly looking for themselves	-	-	-	-	-	-	-	-
Looking for themselves	0.22	0.08	*p* = 0.005 *	0.07; 0.37	0.45	0.11	*p* < 0.001 *	0.23; 0.66
Neutral position	0.50	0.08	*p* < 0.001 *	0.35; 0.66	0.77	0.10	*p* < 0.001 *	0.57; 0.98
Helpful	0.76	0.10	*p* < 0.001 *	0.56; 0.95	0.97	0.14	*p* < 0.001 *	0.69; 1.24
Mostly helpful	1.13	0.19	*p* < 0.001 *	0.76; 1.50	1.33	0.24	*p* < 0.001 *	0.87; 1.79
Trust in politicians								
Mostly untrustworthy	-	-	-	-	-	-	-	-
Untrustworthy	0.18	0.06	*p* = 0.001 *	0.07; 0.29	0.42	0.08	*p* < 0.001 *	0.26; 0.58
Neutral position	0.58	0.06	*p* < 0.001 *	0.46; 0.70	0.86	0.09	*p* < 0.001 *	0.69; 1.04
Trustworthy	0.91	0.14	*p* < 0.001 *	0.63; 1.19	1.31	0.19	*p* < 0.001 *	0.93; 1.68
Mostly trustworthy	1.36	0.43	*p* = 0.002 *	0.52; 2.21	1.63	0.44	*p* < 0.001 *	0.76; 2.49
Trust in the legal system								
Mostly untrustworthy	-	-	-	-	-	-	-	-
Untrustworthy	0.31	0.07	*p* < 0.001 *	0.18; 0.45	0.22	0.11	*p* = 0.026 *	0.03; 0.41
Neutral position	0.76	0.07	*p* < 0.001 *	0.63; 0.90	0.56	0.10	*p* < 0.001 *	0.37; 0.75
Trustworthy	1.10	0.09	*p* < 0.001 *	0.93; 1.27	0.82	0.12	*p* < 0.001 *	0.58; 1.07
Mostly trustworthy	1.10	0.16	*p* < 0.001 *	0.79; 1.42	1.71	0.22	*p* < 0.001 *	1.27; 2.15

* statistically significant.

**Table 8:** Model 4—social trust adjusted for stratification variable.

Predictors	β	SE	*p*	95% CI
Socio-Demographic Characteristics Female (0 = male)	−0.15	0.04	*p* < 0.001 *	−0.22; −0.08
Age	0.01	≈0	*p* = 0.001 *	0.01; 0.01
People trustworthiness				
Mostly careful	-	-	-	-
Careful	0.10	0.06	*p* = 0.084	−0.01; 0.21
Neutral position	0.38	0.06	*p* < 0.001 *	0.27; 0.49
Confident	0.60	0.08	*p* < 0.001 *	0.45; 0.74
Mostly confident	0.35	0.14	*p* = 0.011 *	−0.08; 0.63
People helpfulness				
Mostly looking for themselves	-	-	-	-
Looking for themselves	0.31	0.06	*p* < 0.001 *	0.19; 0.44
Neutral position	0.62	0.06	*p* < 0.001 *	0.49; 0.74
Helpful	0.84	0.08	*p* < 0.001 *	0.68; 1.00
Mostly helpful	1.18	0.15	*p* < 0.001 *	0.89; 1.47
Trust in politicians				
Mostly untrustworthy	-	-	-	-
Untrustworthy	0.27	0.05	*p* < 0.001 *	0.18; 0.36
Neutral position	0.69	0.05	*p* < 0.001 *	0.59; 0.79
Trustworthy	1.07	0.11	*p* < 0.001 *	0.84; 1.30
Mostly trustworthy	1.54	0.30	*p* < 0.001 *	0.95; 2.13
Trust in the legal system				
Mostly untrustworthy	-	-	-	-
Untrustworthy	0.28	0.06	*p* < 0.001 *	0.17; 0.39
Neutral position	0.69	0.06	*p* < 0.001 *	0.58; 0.80
Trustworthy	1.00	0.07	*p* < 0.001 *	0.86; 1.14
Mostly trustworthy	1.51	0.13	*p* < 0.001 *	1.10; 1.61
Stratification				
2002–2010	-	-	-	-
2012–2018	0.72	0.04	*p* < 0.001 *	0.65; 0.79

* statistically significant; R^2^ = 0.129; F = 315.83 (*p* < 0.001).

**Table 9:** Model 5—self-interest.

Predictors	2002–2010 R^2^ = 0.036; F = 93.41 (*p* < 0.001)				2012–2018 R^2^ = 0.018; F = 26.20 (*p* < 0.001)			
	β	SE	*p*	95% CI	β	SE	*p*	95% CI
Socio-Demographic Characteristics Female (0 = male)	−0.07	0.04	p = 0.109	−0.16; 0.02	−0.22	0.07	*p* = 0.001 *	−0.36; −0.09
Age	≈0	≈0	*p* = 0.209	0.00; 0.01	0.01	≈0	*p* < 0.001 *	0.01; 0.01
Education (0 = low education)								
Not possible to harmonize ES-ISCED	-	-	-	-	No data available			
Less than/lower secondary education	0.97	0.05	*p* < 0.001 *	0.88; 1.07	-	-	-	-
Secondary education	0.78	0.09	*p* < 0.001 *	0.61; 0.95	0.20	0.09	*p* = 0.025 *	0.03; 0.37
Tertiary education	0.99	0.10	*p* < 0.001 *	0.78; 1.19	0.51	0.10	*p* < 0.001 *	0.32; 0.70
Health Status Subjective general health								
Good/Very good	-	-	-	-	-	-	-	-
Fair	−0.15	0.05	*p* = 0.005 *	−0.25; −0.04	−0.17	0.08	*p* = 0.034 *	−0.32; −0.13
Bad/Very bad	−0.45	0.07	*p* < 0.001 *	−0.59; −0.30	−0.88	0.12	*p* < 0.001 *	−1.11; −0.65

* statistically significant.

**Table 10:** Model 5—self-interest adjusted for stratification variable.

Predictors	β	SE	*p*	95% CI
Socio-Demographic Characteristics Female (0 = male)	−0.12	0.04	*p* = 0.001 *	−0.20; −0.04
Age	0.01	≈0	*p* < 0.001 *	0.01; 0.01
Education (0 = low education)				
Not possible to harmonize ES-ISCED	-	-	-	-
Less than/lower secondary education	0.90	0.05	*p* < 0.001 *	0.80; 0.99
Secondary education	0.92	0.07	*p* < 0.001 *	0.79; 1.05
Tertiary education	1.21	0.08	*p* < 0.001 *	1.06; 1.36
Health Status Subjective general health				
Good/Very good	-	-	-	-
Fair	−0.16	0.04	*p* < 0.001 *	−0.25; −0.07
Bad/Very bad	−0.59	0.06	*p* < 0.001 *	−0.72; −0.47
Stratification				
2002–2010	-	-	-	-
2012–2018	0.13	0.05	*p* = 0.004 *	0.04; 0.22

* statistically significant; R^2^ = 0.046; F = 153.34 (*p* < 0.001).

## Data Availability

Not applicable.
